# Paracetamol in Pregnancy: Reassurance Amidst Uncertainty

**DOI:** 10.1111/1471-0528.70129

**Published:** 2026-01-05

**Authors:** Athina Samara, Asma Khalil

**Affiliations:** ^1^ Department of Women's and Children's Health Karolinska Institutet Stockholm Sweden; ^2^ Astrid Lindgren Children's Hospital Karolinska University Hospital Stockholm Sweden; ^3^ FUTURE, Center for Functional Tissue Reconstruction, Department of Biomaterials University of Oslo Oslo Norway; ^4^ Fetal Medicine Unit, St George's University Hospitals NHS Foundation Trust University of London London UK; ^5^ Vascular Biology Research Centre, Molecular and Clinical Sciences Research Institute City St George's University of London London UK

**Keywords:** acetaminophen, fetal medicine, fetal physiology, medical disorders in pregnancy, paracetamol

## 1 Introduction

The moment a woman becomes pregnant, the simple act of reaching for a painkiller or antipyretic becomes a dilemma, complicated by scarce alternatives well documented to be worse for babies, such as NSAIDs and narcotics. Yet headaches are common in early pregnancy, migraines often change in pattern [1, 2], and pelvic pain becomes increasingly prevalent as gestation progresses [3], making the need for safe, effective symptom relief both physiological and unavoidable. The result is not informed but constrained choice, landing heavily on those already carrying the biological risks of pregnancy, the clinical burden of symptom control, the psychological strain of uncertainty and the social pressures of caregiving and work.

## 2 Choice Under Constraint

Here we argue that the current discourse, policies and research norms erase real choice for pregnant women because they constrain pregnancy‐appropriate, evidence‐based treatment for pain and fever, pushing women to endure symptoms that carry maternal–foetal risks or to choose between suffering and stigma. In addition, they communicate risk in alarmist or ambiguous ways, amplifying anxiety and decisional conflict while offering few practical alternatives and therefore turning a scientific discourse into a health stressor. Finally, they shift the costs of scientific limitations (not genuine uncertainties) onto vulnerable pregnant women rather than the systems and institutions, expecting them to navigate scarce data, mixed guidance and access barriers on their own, so structural delays become a personal liability.

## 3 Maternal Stress as a Morbidity

Raising unwarranted concern about the safety of using paracetamol in pregnancy is likely to cause stress to many women who have been using it or need to use it for pain relief. This concern is not about paracetamol treating stress, but rather about how excessive or unclear safety messaging itself can create additional psychological burden. Stress is harmful and its weight in pregnancy is chronically under‐recognised. Persistent anxiety and depressive symptoms, financial strain, sociodemographic and structural inequity affect the maternal morbidity index [4, 5]. The CDC has documented that stressful life events in the year preceding childbirth are strongly associated with adverse maternal and infant outcomes, highlighting stress as a public health concern in perinatal care [6]. Similarly, MBRRACE‐UK reports consistently identify maternal mental health problems, including stress‐related disorders, as significant contributors to both maternal morbidity and mortality [7, 8]. These findings showcase the need for systematic screening and timely psychosocial interventions to mitigate the impact of maternal stress on health outcomes.

Pregnancy increases the risk for many causes of headache, including pathologic vascular processes; for example, migraine is the most common cause of headache during pregnancy, often requiring safe symptomatic management. Experiencing unmanaged pain or fever during pregnancy may elevate maternal stress levels, which in turn have been associated with adverse obstetric outcomes including preterm birth, low birth weight and neonatal morbidity [9, 10, 11]. Women who experienced previous perinatal loss also face a significantly higher risk in the subsequent pregnancy [12]. Chronic stress is also linked to worse sleep, higher pain sensitivity, hypertension, gestational complications and adverse perinatal outcomes, including preterm birth and low birth weight [9, 13]. It can amplify nausea and vomiting, trigger or worsen migraines [14, 15, 16] and lower the ability to work or care for other children. Studies have also suggested a link between maternal and prenatal stress and neurodevelopment [17, 18, 19, 20, 21] and conversely, maternal psychological resilience during pregnancy was recently associated with better infant neuropsychological development [22].

The point is not that paracetamol alleviates stress, but that unclear or alarmist communication about its use may heighten maternal anxiety when pain or fever remain untreated. When the system offers few safe, accessible symptom management options and messages that exaggerate risk without acknowledging suffering, it effectively asks women to absorb the risk as stress. Risk communication is essential and should be honest to be empowering; otherwise, it can be weaponised into fear. The line between precaution and alarmism is further crossed when we imply that enduring significant pain or fever is morally superior to taking a well‐understood medication under clinical guidance. That implication is not neutral and redirects risk to women's health and daily functioning.

Compounding this reality, many pregnancies are unplanned. A woman may have taken a medicine before she even knew she was pregnant, then confront guilt and conflicting online advice. What is needed in that moment is clear, proportionate guidance and access to appropriate options, rather than messages that imply uncertainty or blame around treatment decisions.

## 4 Maternal Stress Impact Is Clear, While Paracetamol Association Is Contested

The literature on prenatal paracetamol is heterogeneous in exposure definitions and study designs (Table 1) and a recent review [23] ignited high profile announcements. Meta‐analytic “ever‐use” contrasts and high‐intensity/internal‐dose measures (e.g., cord‐biomarker or meconium studies) often report small‐to‐moderate associations with ASD/ADHD, particularly with frequent or prolonged use, echoing the recent FDA Notice [24, 25, 26, 27, 28, 29]. Smaller biomarker‐based studies report stronger associations but are underpowered, potentially affected by selection bias, and inconsistent with the large cohort findings. Notably, across studies, major uncertainties included important parameters, namely incomplete maternal medication recall, imprecise exposure timing, frequently unmeasured confounders (e.g., maternal infection, psychiatric illness, smoking, BMI), loss to follow‐up and incomplete capture of diagnoses in registries. Thus, while associations have been described, current evidence does not establish causality.

**TABLE 1:** Overview of reports on prenatal paracetamol exposure and autism spectrum disorder (ASD), presented in order of sample size (adapted from Prada et al. Table 2).

Study	*N*	Main finding	Strengths	Limitations	Missing data	Risk interpretation
Ahlqvist et al. 2024 (Sweden, national cohort)	2.48 million	HR 1.05 (1.02–1.08)	Large size, registry linkage	Exposure misclassification, residual confounding	Maternal medication reports incomplete	Very small risk increase; may reflect residual bias more than true effect
Ahlqvist et al. 2024 (Sibling‐controlled)	16 267 siblings	HR 0.98 (0.93–1.04)	Controls for familial/genetic factors	Lower power	Exposure recall still incomplete; imputation used	Null finding weakens causal claim, suggests prior associations confounded
Alemany et al. 2021 (European meta‐cohort)	73 881	OR 1.19 (1.07–1.33)	Multi‐country, prospective	Heterogeneous exposure definitions	Varies by cohort; some loss to follow‐up, incomplete covariates	Suggests modest risk; heterogeneity & missing data limit certainty
Liew et al. 2016 (Denmark)	64 322	HR 1.19 (ASD); HR 1.51 (hyperkinetic symptoms)	National registry, prospective	Self‐report exposure, residual confounding	Self‐report incomplete; covariates (e.g., infection) not fully captured	Consistent with Alemany; unclear dose–response & doubtful causality
Leppert et al. 1999 (UK, Avon)	7786	RR 0.76 (0.51–1.13)	Rich covariate data	Wide CI, null/negative	Some attrition, incomplete follow‐up	No signal; consistent with no causal effect
Avella‐Garcia et al. 2016 (Spain)	1382	IRR ADHD 1.25, impulsivity 1.41	Prospective	Modest size, multiple endpoints	Loss to follow‐up, incomplete confounder adjustment	Mixed behavioural outcomes. Weak evidence
Ji et al. 2020 (Boston, biomarkers)	996	OR 2.14–3.62 (higher tertiles)	Biomarker‐based exposure	Small N, multiple comparisons	Biomarkers measured for subset only; selection bias possible	Stronger signal but underpowered
Saunders et al. 2019 (Canada, case–control)	215	Null result	Case–control contrast	Very small, retrospective recall	Recall bias; incomplete matching	No support for association; weak evidence

*Note:* Listing findings, strengths/limitations, exposure completeness, covariate gaps and risk interpretation. For each report we summarised the main finding, study strengths, limitations, exposure completeness, covariate gaps and a risk interpretation. Overall, associations have been described, but causality remains unproven.

Abbreviations: ASD, autism spectrum disorder; HR, hazard ratio; IRR, incidence rate ratio; OR, odds ratio; RR, risk ratio.

However, large sibling‐control cohorts, which control for shared familial/genetic factors, consistently find no detectable association, with effect estimates shrinking toward null and no dose–response, indicating that earlier signals may reflect confounding rather than a causal effect. The largest national registry cohorts, such as those from Sweden and Denmark, report only very small increases in risk, which may be accounted for by residual confounding or exposure misclassification. When sibling‐controlled designs are applied, the population‐level associations largely attenuate to null, indicating that earlier signals are likely driven by shared familial or genetic factors (Table 2). The large Swedish registry study further shows that any apparent risks in general models disappeared in the full‐sibling analyses [27]. Similarly, the Norwegian Mother and Child Cohort Study results, regarding potential neurodevelopmental sequelae of prenatal paracetamol exposure, showed that signals persisted only with prolonged maternal use (≈ ≥ 28–29 days), while shorter or unspecified durations showed no effect in within‐family comparisons [30, 31]. These results reinforce the conclusion that most observed associations are better explained by residual confounding, particularly shared familial or genetic factors, rather than by a direct effect of paracetamol.

**TABLE 2:** Sibling‐control studies of prenatal paracetamol exposure.

Study (Cohort/Country)	Sample	Exposure ascertainment	Exposure definition	Outcomes	Findings in sibling‐control models	Interpretation
Brandlistuen et al. 2013 (MoBa, Norway)	Sibling subset: ~2919 sibling pairs (same‐sex, exposure‐discordant)	Maternal self‐report (MoBa questionnaires at ~17 weeks, 30 weeks, postpartum)	Duration: 1–27 days versus ≥ 28 days	Child developmental outcomes (gross motor, communication, behaviour)	≥ 28 days exposure linked to poorer motor & communication skills and more behavioural problems versus unexposed siblings; shorter use weaker/null	First within‐family signal; concern mainly for prolonged exposure
Gustavson et al. 2021 (MoBa, Norway)	Full cohort: ~112 973 children; Sibling subset: ~36 516 children from 18 907 families	Maternal self‐report (MoBa pregnancy questionnaires at 17 and 30 weeks)	Cumulative days: ≤ 28 vs. ≥ 29 days	ADHD diagnosis (national registry)	≤ 28 days: no increased ADHD risk; ≥ 29 days: modestly higher ADHD risk in sibling analyses	Replicates the prolonged‐use signal; no effect for shorter use
Ahlqvist et al. 2024 (Sweden, nationwide registry)	Full cohort: 2.48 million children; Sibling subset: 16267 full‐sibling pairs	Antenatal care records (midwife interview) + prescription registers (OTC under‐captured)	Any reported use (yes/no), no precise duration	Autism, ADHD, intellectual disability	No association in sibling‐control analyses; population‐level signals disappeared	Strong evidence that population associations largely reflect familial confounding

In conclusion, while associations have been observed, causality is not established. Notably, a recently published study using four population birth cohorts (US, UK, Ireland, Australia) demonstrated that early‐ and late‐diagnosed autism follow different developmental paths and are tied to different polygenic profiles, highlighting substantial etiologic heterogeneity.

Overall, given the frequent missing/imputed data, variable exposure definitions and inconsistent findings, pregnant women should be reassured that paracetamol remains the recommended first‐line option. Notably, FDA has proposed label changes while UK and EU regulators advise no change and continue to recommend paracetamol when clinically indicated. By contrast, evidence linking maternal fever, migraine and psychosocial stress to adverse outcomes is stronger, more consistent and biologically plausible (Table 3).

**TABLE 3:** Evidence on paracetamol vs. fever, migraine and maternal stress in pregnancy.

Factor	Evidence base	Key limitations	Consensus on causality
Paracetamol (prenatal exposure)	Observational studies, meta‐analyses; some biomarker studies suggest associations with ADHD/ASD	Heterogeneous definitions of exposure, recall bias, confounding by indication (fever/illness), inconsistent dose–response, null findings in sibling‐control designs	Association described, Causal link unproven
Fever/Hyperthermia	Strong epidemiological evidence (meta‐analyses, case–control, cohort studies); biological plausibility (heat disrupts cell division, placental function)	Mainly observational, but consistent across studies and exposures (fever, infection, heat stress)	Causality likely (linked to miscarriage, neural tube defects, preterm birth, stillbirth)
Migraine in pregnancy	Large cohort and case–control studies; migraine linked to preeclampsia, preterm birth, low birthweight, stillbirth	Some heterogeneity by migraine type/severity; fewer intervention trials	Causality plausible (vascular and stress‐related mechanisms)
Maternal stress/anxiety/depression	Consistent associations in meta‐analyses with preterm birth, low birthweight, adverse neurodevelopment	Confounding by socioeconomic and lifestyle factors, but biological mechanisms well‐described (cortisol, inflammation, HPA‐axis)	Causality plausible to likely

## 5 Paracetamol in Pregnancy: Guidelines for Practice

Recent statements from the EMA, MHRA, RCOG, Health Canada and South African professional societies converge, supporting that the use of paracetamol remains the first‐line analgesic/antipyretic in pregnancy, at the lowest dose and for the shortest duration. At the same time, routine medication for low‐grade, short‐lived fevers can often be avoided [32, 33, 34, 35, 36, 37]. This balances maternal comfort and safety with established foetal risks from NSAIDs, which are contraindicated after 20 weeks.

In short, pregnant women should minimise use for routine, low‐grade fevers, treat significant pain or fever with paracetamol at the lowest effective dose for the shortest duration, and avoid NSAIDs in later gestation.

## 6 Even Less Focus on Unplanned Pregnancies

A large share of analgesic exposure occurs before pregnancy is diagnosed, but most debates ignore this reality. Pregnancy is typically recognised at ~5–6 weeks' gestation on average, with a substantial minority recognising at ≥ 7–8 weeks, well into the first trimester. During this interval, many continue their usual self‐care for headaches, colds, dental pain or musculoskeletal pain [38, 39]. A US case–control surveillance study showed NSAID use peaks before conception and drops after recognition, and reported use fell from ~24%–30% in the month before pregnancy to ~10%–11% by the third month [40]. Earlier studies have also shown that first trimester NSAID exposure estimates vary widely by country and data source, ranging from 4.5% based on prescription redemption in Norway to 22.6% in US self‐report datasets, reflecting differences in over the counter (OTC) availability, ascertainment and underreporting [41, 42].

Under‐reporting is likely substantial. When women were specifically prompted about OTC analgesics at the first antenatal visit, 83.7% of those who used any OTC analgesic reported first‐trimester use, implying that much exposure occurs before or very early after recognition [43]. In addition, some OTCs are not considered medication as they are so commonly used (often > 50%–60% in population surveys [44]) and may be omitted unless asked directly, which could further bias early‐pregnancy exposure estimates downward.

## 7 Fever in Pregnancy: Risks and Management

Fever in pregnancy is common, clinically consequential and too often undertreated.

Maternal hyperthermia is associated with miscarriage, congenital anomalies, notably neural tube defects, preterm labour, stillbirth, neonatal sepsis and neurodevelopmental sequelae [45, 46, 47]. These effects are biologically plausible, as heat stress disrupts protein folding, cell division and placental function. Criteria for intra‐amniotic infection, meta‐analyses and climate–health studies also converge on the conclusion that temperature itself can harm maternal and perinatal outcomes [48, 49, 50]. Antipyresis is therefore essential care, not a comfort measure.

## 8 Rebalancing Risk and Responsibility

Systemic gaps, including limited pregnancy‐specific options, fragmented and underpowered dosing data and alarmist communication, shift the burden onto pregnant people, who are asked to endure more pain and stress, navigate contradictory advice and assume responsibility for evidence that has not yet been generated. Transferring risk to those who are already vulnerable is not what precaution or informed choice should mean.

Rebalancing requires investment in the research and tools that create safe alternatives. This means guidance that acknowledges real suffering and success metrics grounded not in communications targets but in evidence‐based care that helps prevent adverse pregnancy outcomes. The choice should not be between a pounding migraine and a pang of guilt but between adequate, data‐supported options, each with clear risks and benefits, and a clear pathway to care.

Being responsible about maternal health means ensuring pregnant people are not deprived of choice by fearmongering. We need to fund the studies, build the registries, design and test new drugs, monitor results and keep guidance updated. Until new options arrive, paracetamol remains the recommended safe first‐line option in pregnancy and we should communicate this with clarity and compassion, honouring the evidence we have and the people who must live with the systemic information gaps. That is what real choice looks like.

## Author Contributions

A.S. and A.K. both conceived and designed the study, conducted the literature search and data extraction, contributed to data verification and interpretation, drafted, critically revised and approved the final manuscript.

## Funding

The authors have nothing to report.

## Conflicts of Interest

The authors declare no conflicts of interest.

## Data Availability

The data that support the findings of this study are available on request from the corresponding author. The data are not publicly available due to privacy or ethical restrictions.
